# Essential Oil and Supercritical Carbon Dioxide Extract of Grapefruit Peels Formulated for *Candida albicans* Infections: Evaluation by an *in Vitro* Model to Study Fungal–Host Interactions

**DOI:** 10.1021/acsomega.2c04189

**Published:** 2022-10-13

**Authors:** Burcu Yaldiz, Pelin Saglam-Metiner, Betul Cakmak, Elif Kaya, Buse Deliogullari, Ozlem Yesil-Celiktas

**Affiliations:** † Department of Bioengineering, Faculty of Engineering, Ege University, 35100, Izmir, Turkey; ‡ Biomedical Technologies Graduate Programme, Graduate School of Natural and Applied Sciences, Ege University,35100 Bornova, Izmir, Turkey

## Abstract

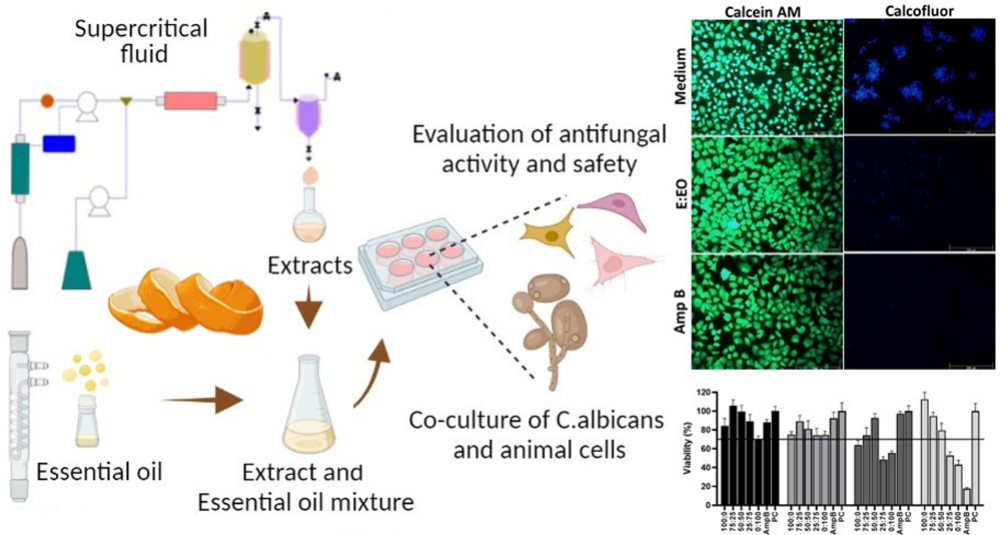

Resistance to currently available antifungal agents raises the need to develop alternative remedies. *Candida albicans* is the most common opportunistic pathogenic fungus of humans, colonizing in the genital and intestinal mucosa, skin, and oral-nasal cavity and reducing quality of life. Herein, essential oil from grapefruit (*Citrus paradise*) peels was obtained by hydrodistillation, and the remaining plant material was sequentially subjected to supercritical carbon dioxide (SC-CO_2_) extraction to determine the conditions for maximizing phenolic compounds. A statistical design was used to evaluate the effect of temperature (30, 50, 70 °C), pressure (80, 150, 220 bar), and ethanol as a cosolvent (0%, 10%, and 20% v/v). Essential oil and SC-CO_2_ extracts were mixed at various ratios to develop an effective antifungal formulation. Subsequently, fungal infection was modeled by coculturing *C. albicans* with human skin keratinocytes (HaCaT) to mimic dermal mycoses, endothelial cells (HUVEC) to evaluate vascular fate, and cervical adenocarcinoma (HeLa) cells to represent additional genital mycoses. Treatment with essential oil and extract (25:75%) formulation for 8 h exhibited slight cytotoxicity toward HeLa cells, no toxicity toward HaCaT and HUVECs, whereas inhibition of *C. albicans*. Considering the clinical significance, such *in vitro* models are essential to screen potential compounds for the treatment of opportunistic fungal infections.

## Introduction

According to the World Health Organization (WHO), infectious diseases are one of the most common causes of death worldwide. Although viruses or bacteria mainly cause these infections, the incidence of opportunistic fungal infections (mycoses) have shown a remarkable increase.^1^ Among different fungi species, *Candida albicans* is the most common opportunistic pathogenic fungus of humans. While it is a commensal fungus that colonizes the genital and intestinal mucosa, skin, and oral-nasal cavity of 30–70% of healthy individuals,^2,3^ it becomes an opportunistic pathogen in immunologically weakened and immunocompromised individuals.^4^ The pathogenic form of *C. albicans* might cause severe mucosal and systemic infections that significantly reduce the quality of life and is associated with high mortality.^5^ Furthermore, systemic candidiasis has been reported in severe COVID-19-associated pneumonia patients.^6^ Although topical and systemic antifungal agents such as ketoconazole and fluconazole are widely used in *C. albicans* infections, most studies have shown that long-term treatment with these agents may lead to the development of antifungal resistance.^7,8^ Hence, there is an urgent need for the development of safe and efficacious antifungal therapeutic agents.^9^ Today, the use of plant extracts such as essential oil (EO) in the fields of pharmacy and cosmetics has become quite popular due to its antifungal, antioxidant, antiallergic, anticarcinogenic, and anti-inflammatory effects.^10−12^ In particular, EOs obtained from citrus species are reported to have potent antifungal effects against fungal infections, including infections caused by *C. albicans*.^13^ Due to these health beneficial properties of citrus oil, the market is witnessing significant growth and is expected to reach USD 8.49 billion by 2027.^14,15^ In order to meet this demand, hydrodistallation is one of the most preferred techniques. However, the increase in the production of EOs also leads to a significant increase in the amount of waste plant materials that still contains valuable components after distillation. Extraction of phenolic compounds from waste plant materials can offer an innovative opportunity to utilize processing wastes. Some of these phenolic compounds exhibit anti-Candida properties by causing inactivation of enzyme production and exhibiting antibiofilm activity.^16^ It is worth to mention that the methods used to extract phenolic compounds from plant materials play an important role in therapeutic activity, quality, and yield of the final product.^17^ Although various conventional and innovative methods, such as solvent extraction and microwave-assisted extraction, are used for extraction, some drawbacks should be mentioned such as high solvent consumption, inability to completely remove organic solvents from the product, and long extraction times, particularly with solvent extraction. Compared to other extraction methods, supercritical fluid extraction (SFE) enables the extraction of valuable compounds from plant based materials in accordance with the UN Sustainable Development Goals (SDGs), contributing to the circular economy.^18^ As the solvating power of supercritical fluids can be adjusted with small alterations in temperature and pressure, maximizing the extraction of targeted compounds with high purity can easily be achieved. Supercritical CO_2_ (SC–CO_2_), a nonpolar solvent, is frequently used in SFE owing to the low critical temperature (31 °C), which prevents thermal degradation of heat-sensitive compounds.^19^ However, when SFE is applied to extract polar compounds, ethanol is introduced to the system as a cosolvent to increase polarity, since CO_2_ is nonpolar and cannot dissolve polar compounds such as phenolic compounds.^20^ In this study, EO from grapefruit peels were obtained by hydrodistillation, and the remaining plant material was sequentially subjected to SC–CO_2_ extraction to obtain phenolic compounds. We hypothesized that if EO and SC–CO_2_ extracts were mixed at a certain ratio, then an effective antifungal formulation can be developed. Subsequently, fungal infection was modeled by coculturing *C. albicans* with human skin keratinocytes (HaCaT), human umbilicord vein endothelial (HUVEC), and cervical adenocarcinoma (HeLa) cells to validate the antifungal effect of the formulation. To infect the host, *C. albicans* adheres to the host cells, colonizes, is subjected to morphogenesis, followed by tissue invasion, vascular dissemination, and colonization.^21^ To the best of our knowledge, this is the first study formulating essential oil with the extract of the peel and testing the efficacy against *C. albicans* cocultured with skin keratinocytes to mimic dermal mycoses, endothelial cells to evaluate vascular fate, and cervical cancer cells to represent additional genital mycoses.

## Materials and Methods

### Plant Material

Ripe grapefruits were obtained from the Aegean region of Turkey. The peels from grapefruits were dried at 40–50 °C in the oven for about 2 days and stored at −20 °C for further use.

### Essential Oil Distillation of Grapefruit Peels

Dried grapefruit peels were ground (<2 mm) with a blender (Waring), and 50 g was weighed and added into a volumetric flask containing 500 mL of distilled water. Prepared samples were distilled by hydrodistillation using a Clevenger apparatus. The distillation time after boiling was determined as 2.5 h at atmospheric pressure. After distillation, the volatile oil accumulated in the Clevenger device was removed to the dark-sealed vial. All distillates were kept at −20 °C until analysis. The remaining plant material from the distillation was then allowed to dry for about 1.5 days in the oven at 40 °C and stored at +4 °C for use in supercritical fluid extraction.

### Gas Chromatography/Mass Spectrometry (GC–MS) Analysis

Volatile compounds were analyzed by a 7890B gas chromatography coupled with a PAL RSI 85 autosampler and MSD 5977A mass spectrometer equipped with capillary HP-INNOWAX (60 m length; 0.320 mm i.d.; 0.25 μm film thickness). Helium was used as the carrier gas at a flow rate of 0.7 mL/min and an ionization voltage of 70 eV. The oven temperature program in GC-MS was from 50 to 210 °C at 4 °C/min for 3 min, and then the programmed temperature rose up 280 °C at 20 °C/min for 5 min. A sample of 1.0 μL was injected in the split mode system with a split ratio of 1:50. Mass spectra were collected in the range of 50–550 atomic mass units (AMU). Each compound was identified by comparing its mass spectra with the mass spectra from the WILEY library. Integrations were made with MassHunter software. Concentrations of compounds were calculated from the peak areas and shown as percentages.

### Supercritical Fluid Extraction of Grapefruit Peels

Supercritical carbon dioxide extraction was carried out on an SFE 100 System (Thar Instruments, Inc., UK, 2006) (Figure S1). The extractor volume was 100 mL and was filled with 25 g of remaining plant material from distillation. Optimization was designed using the Box–Behnken program. The parameters for extraction were temperature (32, 50, 70 °C), pressure (80, 150, 220 bar), and cosolvent ratio (0%, 10%, and 20%, v/v), while other variables were kept constant as 10 g/min flow rate and 60 min extraction time. Once the desired temperature was reached in each experiment, CO_2_ was pumped into the extraction vessel until the process pressure was reached. When the extraction was complete, depressurization of the extraction column was performed, and then the extracts were collected from the separation vessel. Solvent was evaporated by a rotary vacuum evaporator, and samples were stored in the dark at +4 °C for further analysis.

### Statistical Design

Response surface methodology (RSM) which is an optimization approach consisting of a set of mathematical and statistical methods^22^ was used to optimize the extraction process. A Box–-Behnken design was utilized to evaluate the effects of three independent variables (temperature, pressure, cosolvent ratio) and their impacts on the total phenol content of the extracts. The special arrangement of the Box–Behnken design levels allows the number of design points to increase at the same rate as the number of polynomial coefficients.^23^ This design suggests how to select points from the three-level factorial arrangement, which allows the efficient estimation of the first- and second-order coefficients of the mathematical model. These designs are more efficient and economical than their corresponding 3k designs, mainly for a large number of variables.^24^

### Determination of Total Phenolic Compounds

The total phenols in the extracts were determined according to the Folin–Ciocalteu method.^25^ About, 0.1 mL aliquot extract was added into a tube reaching a final volume of 10 mL of distilled water prepared by an in-house nanopure water system (Sartorius Arium 611, Sartorius- Stedim, Germany). Subsequently, 0.5 mL of Folin–Ciocalteu’s reagent (Sigma-Aldrich, USA) was added and vortexed. After 5 min, 1.5 mL of sodium carbonate (Sigma-Aldrich, Germany) solution was added, stirred, and left at room temperature for an hour. Absorbance was measured at 760 nm. All of the experiments were performed as duplicates. Gallic acid (Fluka, Germany) was used as a standard, and the results were calculated as gallic acid equivalent (GAE) per gram of extract.

### Radical Scavenging Activity (RSA)

The free radical scavenging activities of extracts were determined as reported previously.^26^ The extracts dissolved in 4 mL of ethanol were added to 0.5 mL of 1 mM methanolic solution of 2,2-diphenyl-1-picrylhydrazyl hydrate (DPPH) (Sigma-Aldrich, USA). The contents were stirred for 15 s and then left at room temperature in the dark for 30 min. A control sample contained the same amount of ethanol and DPPH solution. The decrease in colorization was measured at 517 nm. Inhibition of the DPPH radical was calculated as follows: DPPH scavenging activity (%): [(*A* – *B*)/*A*] × 100 where *A* is the absorbance of the control, and *B* is the absorbance of the extract. All of the experiments were carried out in duplicate.

### Animal Cell and Yeast Cultures

Human cervical adenocarcinoma (HeLa) cells, human umbilical vein endothelial (HUVEC) cells, and human skin keratinocyte cell line (HaCaT) cells were obtained from the American Cell Culture Collection (ATCC). HeLa and HUVEC cells were maintained in RPMI 1640 basal medium supplemented with 10% fetal bovine serum (FBS), 1% l-glutamine (200 mM), 1% nonessential amino acid (NEAA), 1% sodium pyruvate, and 0.1% gentamicin (10 mg/mL). HaCaT cells were maintained in Dulbecco’s modified Eagle’s medium-high glucose (DMEM HG) supplemented with 10% FBS, 1% l-glutamine (200 mM), and 0.1% gentamicin (10 mg/mL). All cells were cultured in T-flasks with filtered vent caps (Corning, USA) in a monolayer to 80% confluence in a humidified incubator with 5% CO_2_ at 37 °C and then harvested with 0.25% trypsin-EDTA. All cell culture reagents were supplied by Sigma-Aldrich, USA.

*Candida albicans* used as yeast was obtained from ATCC (10231) and cultured in yeast extract peptone dextrose broth (YPDB) in a shaking flask at 35 ± 2 °C in an incubator. All culture reagents were supplied by Sigma-Aldrich, USA.

### Cytotoxicity of Essential Oil and SC-CO_2_ Extracts

The cytotoxic activities of two grapefruit extracts (E1 and E2), EO and E:EO mixtures on HeLa, HaCaT, HUVEC cell lines, and *C. albicans* were determined by the 2,5-diphenyl-2*H*-tetrazolium bromide (MTT) assay. For cytotoxicity, cells in the exponential growth phase were cultured in 96-well plates at 1 × 10^4^ cells/well at 37 °C, 5% CO_2_ in a fully humidified incubator overnight. After the culture medium was removed, 100 μL of dose-dependent E1, E2 (500, 250, 125, 62.5, 31.25, 15.6, 7.8, 3.9 μg/mL), EO (3200, 1600, 800, 400, 200, 100, 50, 25, 12.5 μg/mL), and E:EO mixtures (ratio of 31.25 μg/mL for E2 and 800 μg/mL for EO at 8 h; 100:0, 75:25, 50:50, 25:75 and 0:100%) were added to each well and incubated for 8 and 24 h as four replicates in an incubator at 37 °C, 5% CO_2_. The cells were tested in growth medium only as a positive control (untreated cells), 100% DMSO-treated cells as a negative control, and ethanol as much as the maximum extract percentage as a solvent control. At the end of the treatment time, the medium was removed, and 10% MTT solution was added to each well and incubated for 3 h at 37 °C in dark. Then, the medium was removed, DMSO was added, and the quantity of formazan product was determined based on the absorbance at 570 nm (BioTek Instruments, Inc., headquartered in Winooski, VT, USA). Percent cell viability was calculated as per the formula (absorbance of adjuvant-treated cells/absorbance of untreated control cells × 100), whereas IC_50_ values were calculated by using GraphPad Prism 7.0.^27^ For *C. albicans* cytotoxicity, 145 μL of dose-dependent E1, E2 (500, 250, 125, 62.5, 31.25, 15.6, 7.8, 3.9 μg/mL), EO (3200, 1600, 800, 400, 200, 100, 50, 25, 12.5 μg/mL) and E:EO mixtures (ratio of 31.25 μg/mL for E2 and 800 μg/mL for EO at 8 h; 100:0, 75:25, 50:50, 25:75 and 0:100%) in RPMI medium were added to each well as four replicates. Subsequently, 5 μL of yeast in the exponential growth phase was added to each well as 1 × 10^5^ yeasts/well, and then incubated for 8 and 24 h at 35 ± 2 °C. The yeast was tested in RPMI medium only as a positive control (untreated yeast), and 2 μM amphotericin B-treated yeast was used as a negative control, 100% DMSO-treated yeast as a DMSO control and ethanol-treated yeast, as much as the maximum extract percentage as solvent control. At the end of the treatment period, the plate was centrifuged, and the medium was removed. Then, 10% MTT solution was added onto the yeasts, and the next steps were done as described above.

### *In Vitro* Model to Study Fungal–Host Interactions

In order to determine the efficiency of E:EO formulations using an *in vitro* fungal infection model, an effective 2D coculture system was established. Briefly, HeLa, HaCaT, and HUVEC cells were seeded in 24-well plates as 1 × 10^5^ cells/well at 37 °C, 5% CO_2_ in a fully humidified incubator overnight. Then, growth medium was replaced with 950 μL of most effective extract:essential oil (E:EO) formulation (25:75%) in RPMI medium, and 50 μL *C. albicans* was cocultured in each well at a concentration of 1 × 10^6^ yeasts/well and incubated for 8 h at 37 ± 2 °C. RPMI medium and 2 μM amphotericin B-treatment were used as positive and negative controls, respectively. After 8 h, Live&Dead (L3224, Molecular Probes, Thermo, USA) and Calcofluor (18909, Sigma-Aldrich, USA) fluorescent staining assays were performed. According to the staining method, half of the culture medium was first carefully removed. Then, 2 μM calcein AM (green) and 4 μM EthD-1 (red) in 1× PBS dye solutions were applied to cells and then incubated at room temperature in the dark. After 30 min, cultures were washed with 1× PBS carefully.^28^ A final concentration of 100 μg/mL Calcofluor dye and one drop of 10% KOH solution were added to coculture (18909, Sigma-Merck, Technical Bulletin Rev 09/2021). Stained cells yeast were examined under a fluorescence microscope (Zeiss, Axial 2.0, Germany). Green dye represents living animal cells, red dye represents dead animal cells, whereas blue is associated with yeasts.

## Results and Discussion

### GC-MS Profile of Essential Oil Extracted from Grapefruit Peel

In this study, EO of grapefruit peels were obtained by hydrodistillation, and then the chemical composition of the EO was analyzed by GS-MS. The hydrodistillation of the dried grapefruit peels gave EO with a yield of 2% (w/w). A total of 131 different components were identified in the EO of grapefruit peel by GS-MS. The major components comprising 88.79% of identified 131 components in the EO are listed (Table 1), along with their retention time and concentration (%)

**Table 1:** GC-MS Profile of EO Extracted from Grapefruit Peels by Hydrodistillation

peak no.	Component	Retention time (min)	Concentration (%)
1	α-pinene	9.2201	0.97
2	hexanal	10.8097	0.01
3	2-β-pinene	11.5829	0.05
5	β-myrcene	13.218	3.13
6	α-terpinene	13.8375	0.05
7	dl-limonene	14.732	79.85
8	sabinene	14.9025	0.72
12	γ-terpinene	15.9973	0.08
15	α-terpinolene	17.2445	0.03
16	octanal (CAS)	17.4017	0.33
17	(*E*)-4,8-dimethyl-1,3,7-nonatriene	17.9294	0.02
18	nonanal (CAS)	20.8611	0.09
19	1,3,8-*p*-menthatriene	20.9927	0.02
20	*cis*-linalool oxide	22.3956	0.76
21	α-cubebene	23.0113	0.02
26	α-copaene	24.1937	0.40
29	β-cubebene	25.5917	0.20
40	naphthalene	29.3343	0.02
122	nootkatone	49.0324	2.04
	total identified (%)		88.79
	other	varies	11.21

The compounds identified were hydrocarbons, alcohols, esters, aldehydes, and terpenes. These compounds are essential and have fragrant aromas in addition to contributing to some medicinal values.^29^dl-Limonene, β-myrcene, nootkatone, α-pinene, *cis*-linalool oxide, and sabinene were among the compounds detected by GC-MS, where dl-limonene (79.85%) was the major component, followed by β-myrcene (3.13%) and noontkatone (2.04%). Limonene is an important citrus compound that has anticancer, anti-inflammatory, and antitumor properties and the background flavor of the grapefruit,^30^ whereas β-myrcene is a monoterpene-built EO component that provides a pleasant smell.^31^ In addition, the presence of nootkatone is noteworthy, which is a conjugated terpene ketone being the most important aromatic organic compound of grapefruit.^32^

### Optimization of Supercritical Carbon Dioxide Extraction of Grapefruit Peels

Supercritical carbon dioxide extraction of the remaining plant material from distillation was optimized to elicit the process conditions maximizing total phenols. The effects of pressure (80, 150, 220 bar), temperature (30, 50, 70 °C), and cosolvent ratios (0%, 10%, and 20%, v/v) were investigated. A second-order polynomial equation was used to express the total phenolic compounds (TPC), Y1 (mg GAE/g extract) as a function of the coded independent variables, where A, B, and C represent the codes of temperature, pressure, and cosolvent ratio in CO_2_, respectively (Table 2).
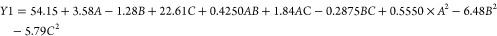


**Table 2:** Experimental Points for Independent Variables, Yield (mg Extract/g Remaining Plant Material), Total Phenolic Compounds (mg GAE/g), and Radical Scavenging Activities (RSA%) of Extracts

	A	B	C		Y1	
samples	*T* (°C)	*P* (bar)	cosolvent (%)	yield (mg/g)	TPC (mg GAE/g)	RSA (%)
**SFE1**	30	150	0	3.97	21.92 ± 0.001	5.72 ± 0.008
**SFE2**	50	220	0	2.97	20.56 ± 0.095	6.99 ± 0.026
**SFE3 (E1)**	70	150	20	10.70	79.60 ± 0.067	44.37 ± 0.003
**SFE4**	50	150	10	9.61	53.40 ± 0.003	39.60 ± 0.025
**SFE5**	50	220	20	16.17	60.03 ± 0.007	56.00 ± 0.135
**SFE6**	30	80	10	7.70	46.10 ± 0.017	57.18 ± 0.003
**SFE7**	50	150	10	8.02	53.43 ± 0.006	41.94 ± 0.001
**SFE8**	50	150	10	7.36	55.65 ± 0.055	73.19 ± 0.042
**SFE9**	30	220	10	6.88	43.30 ± 0.006	50.76 ± 0.010
**SFE10**	70	220	10	4.66	51.20 ± 0.019	71.32 ± 0.029
**SFE11**	70	80	10	3.02	52.30 ± 0.004	48.00 ± 0.021
**SFE12**	70	150	0	2.05	25.52 ± 0.005	13.46 ± 0.051
**SFE13**	50	80	20	8.14	63.78 ± 0.025	41.24 ± 0.008
**SFE14 (E2)**	30	150	20	9.70	68.63 ± 0.029	84.85 ± 0.044
**SFE15**	50	80	0	4.85	23.16 ± 0.004	48.05 ± 0.015

Based on the analysis of variance (ANOVA), the correlation coefficient of the fitted model represented the experimental data well with an *R*^2^ value of 0.9873, and the response (Y1, *p* < 0.05) was statistically significant. The significance of the model and a nonsignificance of the lack of fit indicated that the developed model for TPC yield prediction from grapefruit was a good fit. Therefore, it is possible to find the model equation to estimate the total phenolic compounds using any combination of values of the variables.

The extractability of TPC was extremely low when CO_2_ was applied alone due to the nonpolar nature. This has been reflected on the extraction yields, which varied between 2.97 to 16.17 mg/g, and extract yields were much lower when only CO_2_ was applied compared to those of ethanol entrained CO_2_. Although pressure was not significant (*p* > 0.05), temperature and cosolvent ratios were found statistically significant (*p* < 0.05) (Table 3). Thus, TPC contents were sensitive even to the minor alterations of temperature and cosolvent ratios. Considering the interaction of temperature and pressure, comparatively higher TPC yields were obtained above 50 °C at the pressure zone of 80–150 bar (Figure S2A). Limonoid glycosides were extracted from grapefruit mollases using supercritical fluid extraction, and the optimized conditions were reported as 483 bar, 50 °C and 10% ethanol.^33^ In regards to the relation between pressure and cosolvent, higher yields were attained at cosolvent ratios above 15% (Figure S2B) as a result of enhanced interactions between the matrix and the polar cosolvent, which induces changes in the structure of the cellular matrix via intracrystalline and osmotic swelling and break analyte-matrix bindings by competing with polar interactions between matrix and the compounds to be extracted.^34^ Considering the relation between temperature and cosolvent, high yields were observed above 50 °C using a cosolvent ratio between 15 and 20% (Figure S2C). Although the solvating power of SC–CO_2_ depends on its pressure and temperature and the increase in pressure enhances the solubility,^20^ the treatments at 220 bar have not exerted a significant effect on the extraction efficiency. However, the extraction yield was sensitive to the alterations in temperature and cosolvent ratio, where TPC values of about 43–55 mg GAE/g were achieved at 10% (v/v) cosolvent with varying temperature values, whereas TPC values reached about 79 mg GAE/g at 20% (v/v) cosolvent ratio. Overall, two experimental conditions, referred to as E1 and E2 yielded the highest TPC values of 79.60 and 68.78 mg GAE/g, respectively. Both processes were carried out at 150 bar and a cosolvent ratio of 20% (v/v), but the increase in applied temperature, which was 70 °C for E1 and 30 °C for E2, resulted in an increase in the TPC value. The influence of temperature on the solid solubility is the result of two competing effects; the increase of solid volatility and the decrease of solvent density with temperature rise.^34^ If the density effect was predominant, the solubility of the TPC in the supercritical phase would have decreased at higher temperatures. In the case that the vapor pressure is overwhelming, the solubility of the TPC would increase with the increase in the vapor pressure.^35^ The enhanced extractability of phenolic compounds in the grapefruit peel might be associated with the predominance of the vapor pressure effect over the density. In regards to free radical scavenging capabilities, E1 yielded an RSA value of 44.37%, whereas this value was doubled for E2 (84.85%). It is worth to mention that TPC and RSA might not always be directly proportional as reported in some studies, while TPC changed with varying temperature and pressure values, RSA remained the same.^36,37^ As phenolic compounds are responsible for a wide range of biological activities, the antifungal formulations were based on these two extracts, E1 and E2, along with combinations of the EO.

**Table 3:** Analyses of Variance (ANOVA) According to the Box–Behnken Model

source	sum of squares	degrees of freedom	mean square	*F*-value	probability (p) > *F*
model	4486.80	9	498.53	43.19	0.0003
A - temperature	102.75	1	102.75	8.90	0.0307
B - pressure	13.13	1	13.13	1.14	0.3349
C - EtOH conc.	4089.70	1	4089.70	354.31	<0.0001
AB	0.7225	1	0.7225	0.0626	0.8124
AC	13.58	1	13.58	1.18	0.3276
BC	0.3306	1	0.3306	0.0286	0.8722
A^2^	1.14	1	1.14	0.0985	0.7663
*B*^2^	155.04	1	155.04	13.43	0.0145
*C*^2^	123.67	1	123.67	10.71	0.0221
residual	57.71	5	11.54		
lack of fit	54.34	3	18.11	10.73	0.0864
pure error	3.38	2	1.69		
cor total	4544.51	14			
*R*^2^	0.9873				
*R*^2^ adj.	0.9644				

### Cytotoxicity of Essential Oil and SC–CO_2_ Extracts

**Figure 1:**
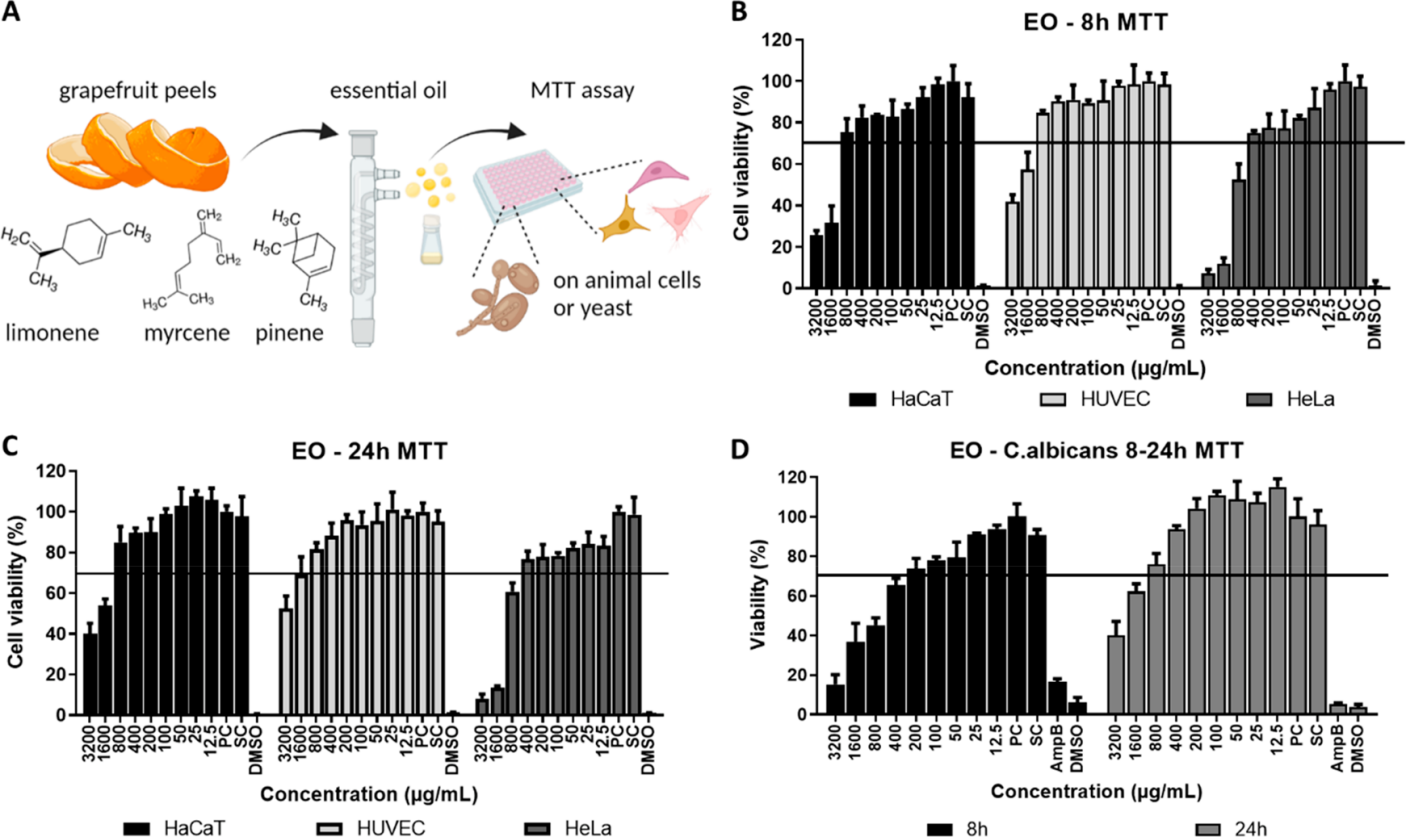
(A) Schematic depiction of the MTT test for EO. Cell viability results (%) of EO on HaCaT, HUVEC, and HeLa cells at (B) 8 h and (C) 24 h, and (D) *C. albicans* yeast at 8–24 h.

The cytotoxic activity of EO obtained by hydrodistillation (Figure 1A) and SC-CO_2_ extracts on HaCaT, HUVEC, HeLa cells, and *C. albicans* were investigated. To be used as an antifungal agent, an EO and extract should be cytotoxic to *C. albicans* without altering the viability of healthy cells such as HaCaT and HUVECs used in this study.

**Figure 2:**
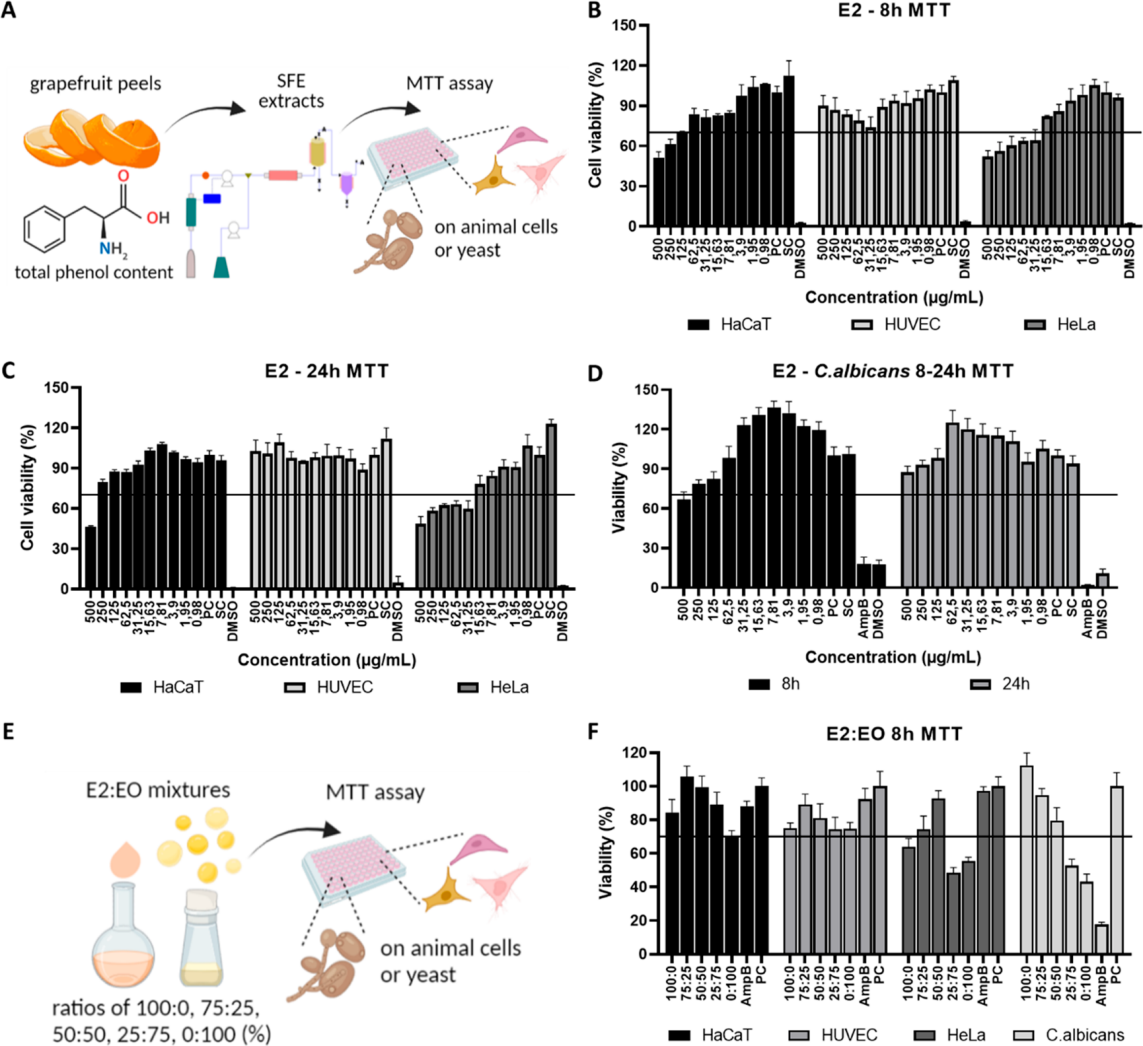
(A) Schematic depiction of MTT test for E2. Cell viability results (%) of E2 on HaCaT, HUVEC, and HeLa cells at (B) 8 h and (C) 24 h, and (D) *C. albicans* yeast at 8–24 h. (E) Schematic depicting MTT test for E2:EO mixtures, (F) viability of E2:EO mixtures on animal cells and yeast at 8 h.

The cell viabilities of EO on both cells were above 70% at concentrations of 800 μg/mL and below incubated for 8 and 24 h. However, EO was cytotoxic to HaCaT and HUVEC cells at concentrations of 3200 and 1600 μg/mL for 8 and 24 h, while cytotoxicity has been observed for HeLa not only at these concentrations but also at 800 μg/mL as well (Figure 1B,C), indicating the selective effect of EO at 800 μg/mL. This profound effect might be associated with the presence of dl-limonene, the major compound (79.85%) in grapefruit EO, as its metabolites peryllic acid and perillyl alcohol exerted cytotoxicity on cancer cells.^38^ In addition to mammalian cells, EO was observed to be toxic to pathogenic *C. albicans* at a concentration range of 3200–400 μg/mL at 8 h, but only at concentrations of 3200 and 1600 μg/mL at 24 h due to its rapid proliferation and resistance to EO (Figure 1D). Similarly, the grapefruit peel EO concentration required for 50% viability of *C. albicans* was shown to be lower than that of healthy epithelial Vero cells, which makes it a natural alternative for the control of mycoses.^39^ Considering the results of our study, 800 μg/mL has been elicited as the ideal concentration of EO to prepare mixtures with SC-CO_2_ extracts and a treatment interval of 8 h, which would further be tested for cytotoxicity. Among SC-CO_2_ extracts, E1 was cytotoxic to all cells and the yeast at a concentration of 250 μg/mL and below at 8 h (Figure S3), showing no selectivity. Thus, E1 was excluded for preparation of E:EO mixtures. On the contrary, E2 extract (Figure 2A) showed slight cytotoxicity to HeLa cells (viability was below 65%) than HaCaT and HUVECs at concentrations above 31.25 μg/mL for both 8 and 24 h (Figure 2B,C). However, no toxic effect of these concentrations was observed on yeast during these hours (Figure 2D). Vihanova et al. compared the antibacterial effect of EOs and SC-CO_2_ extracts of *Cinnamomum spp*. and showed that the EO exhibited higher antibacterial activity than the extract.^40^ As EO reduced both cancer and yeast viability and E2 reduced cancer cell viability while promoting the viability of healthy cells at 8 h, we hypothesized that mixing EO with E2 at various ratios would yield selective activity. While preparing E2:EO formulations, we used E2 at a concentration of 31.25 μg/mL and EO at a concentration of 800 μg/mL at ratios of 100:0, 75:25, 50:50, 25:75, and 0:100 (%). The cytotoxic activities of E2:EO mixtures on HacaT, HUVEC, HeLa cells, and *C. albicans* was evaluated by an MTT assay at 8 h (Figure 2E). None of the tested E2:EO mixtures exhibited any cytotoxicity on HaCat and HUVEC healthy cells as expected, while the 25:75 and 0:100 (%) ratios showed more toxic effects on HeLa cells by reducing viability to 48.6% and *C. albicans* to 52.8% (Figure 2F).

The effect of an antifungal agent amphotericin B (Amp B) was investigated to determine whether culture conditions affect the susceptibility of *C. albicans* to drugs, and the viability was reduced to 17.6%.

### *In Vitro* Model to Study Fungal–Host Interactions

**Figure 3:**
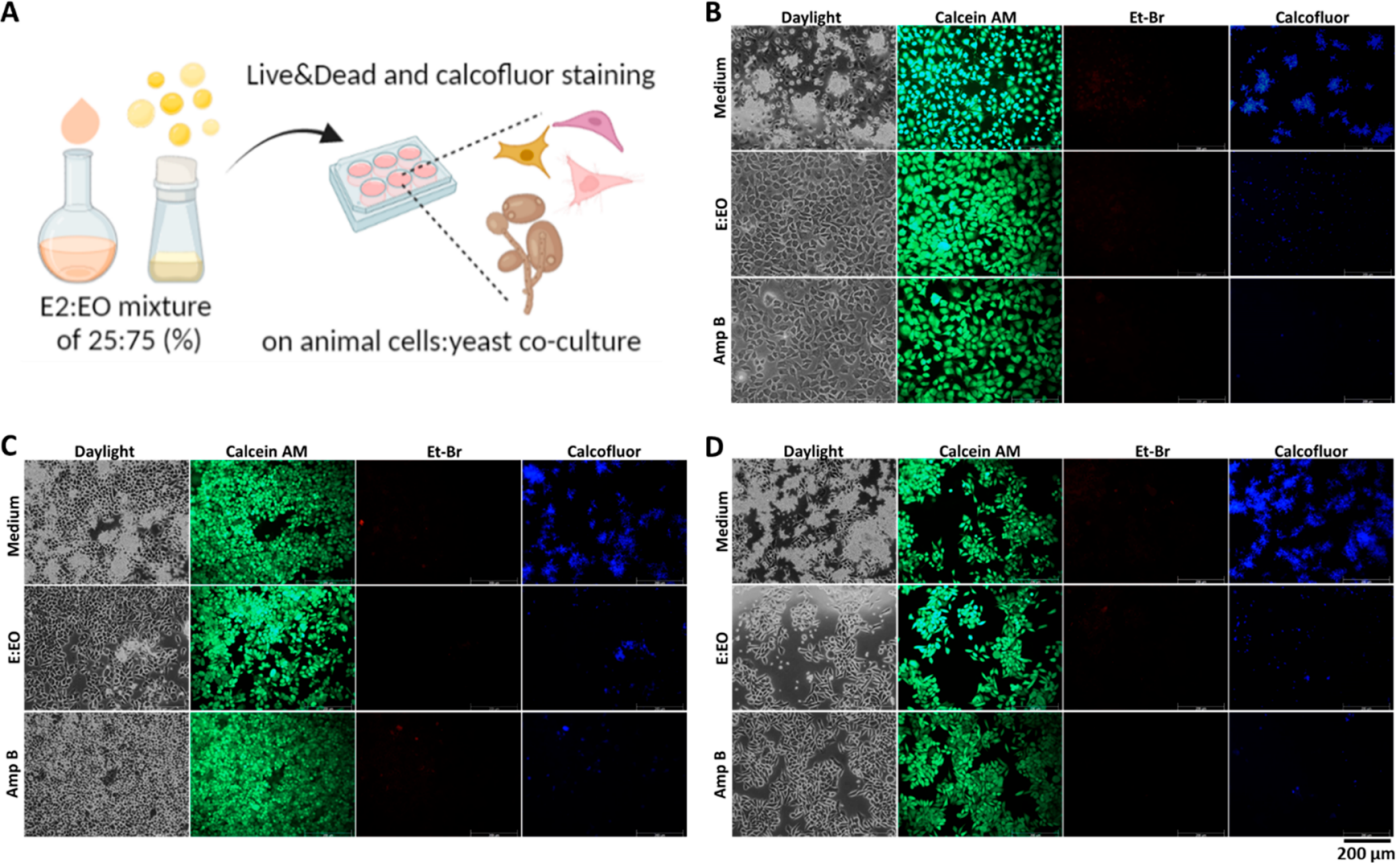
(A) Schematic depicting Live & Dead and calcofluor staining for E2:EO (25:75%). Daylight/fluorescent images of (B) HaCaT, (C) HUVEC, and (D) HeLa cells: *C. albicans* cocultures after E2:EO (25:75%) treatment for 8 h. The green ones are living animal cells, red ones are dead animal cells and blue ones are only living yeasts (bar scale 200 μm).

For treatment of mycoses, we need to understand the complex fungal–host interplay during pathogenesis, the virulence caused by the fungi, and the response of the host to infection by immunological defenses. *In vitro* models can be used to mimic fungal infections of various tissues and organs and the corresponding immune responses at near-physiological conditions. Furthermore, models can include fungal interactions with the host–microbiota to mimic the *in vivo* situation on skin and mucosal surfaces.^41^ In this study, a fungal infection was modeled by coculturing *C. albicans* with human skin keratinocytes (HaCaT), endothelial (HUVEC), and cervical adenocarcinoma (HeLa) cells to validate the antifungal effect of the formulation, E2:EO (25:75%) (Figure 3A) and qualitative light/fluorescent staining (Live & Dead and calcofluor) were performed at 8 h (Figure 3B–D). While intense yeast proliferation suppressing the viabilities of healthy cells (HaCaT and HUVEC) was dominant in untreated cocultures, increased cell viabilities were easily observed after suppressed yeast proliferation in E2:EO treated coculture in comparison to the Amp B treatment which inhibited yeast proliferation. This profound effect might be associated with the presence of limonene, reported to inhibit growth of *C. albicans* by cell wall/membrane damage inducing oxidative stress that causes DNA damage, finally leading to apoptosis.^42^ Furthermore, decreased viability of HeLa cells was observed as well as suppressed *C. albicans* proliferation after 8 h of E2:EO treatment applied to HeLa cells, which led to further reductions in fungal and cancer cells. There are various studies showing that EOs or extracts obtained from different plant sources exhibit antifungal effects on *C. albicans* or HeLa cancer cells, but do not exert cytotoxic effects on healthy cells such as HaCaT or HUVEC^43−45^ as well as the effect of *C. albicans* on the viability of oral mucosal epithelial cells.^46^ However, the combination of *C. albicans* and other mammalian cells has not been investigated in these studies. Therefore, this *in vitro* model sufficiently represents the fungal–host interaction, which is also validated by applying a known antifungal agent, Amp B. Additionally, E2:EO formulated as 25:75% has proved to be effective based on calcofluor staining, significantly inhibiting *C. albicans* especially in HaCaT and HeLa cells, indicating the applicability of the formulation in dermal and genital mycoses. However, the colonization has not been completely inhibited in endothelial cells. The first point of contact between *C. albicans* and the endothelium is the outer layer containing proteins and carbohydrates, which are reported to have a number of functions, including the ability to act as adhesion molecules, proteins with integrin-like properties, Candida agglutinin-like sequence gene products^47^ and mannans.^48^ More effective therapeutic regimens are required for inhibition of vascular dissemination and endothelial colonization.

## Conclusion

EOs and extracts containing *phenolic compounds* have gained immense attention due to antifungal, antimicrobial, anticancer, and antioxidant properties. In this study, the potential of EO and extracts obtained from grapefruit peels by hydrodistillation and SC-CO_2_ extraction was investigated to provide an effective and safe treatment approach for *C. albicans* infections. The treatment with the mixture of extract and EO (25:75%) for 8 h showed effective antifungal and anticancer activities on *C. albicans* and HeLA, respectively. In addition, the formulation has proven to be safe by promoting the viability of healthy mammalian cells HUVEC and HaCaT. The findings particularly highlighted the antifungal and anticancer therapeutic value of the EO and extract from grapefruit peel, demonstrating the potential usability in fungal infections. Additionally, the developed *in vitro* model with human keratinocytes and endothelial cells cocultured with *C. albicans* recapitulated the interaction between the host and the fungus. Considering the clinical significance, such *in vitro* models are essential to screen various compounds or combinations for the treatment of opportunistic fungal infections.

## Supporting Information Available

The 3D response surface plots of total phenol contents showing the effects of temperature and pressure at constant optimum cosolvent (20%), pressure, and cosolvent at constant temperature (70 °C), temperature and cosolvent ratio at constant pressure (150 bar). Cell viability results (%) of the formulation on human skin keratinocytes (HaCaT), human umbilicord vein endothelial (HUVEC), cervical adenocarcinoma (HeLa) cells, and *C. albicans* yeast at 8–24 h (PDF)

## Abbreviations

E1/E2: extract
EO: essential oil
SFE: supercritical fluid extraction
SC-CO_2_: supercritical CO_2:_
HaCat: human skin keratinocytes
HUVEC: human umbilical cord vein endothelial
HeLa: cervical adenocarcinoma
(GC-MS): gas chromatography/mass spectrometry
