# Supplementary material for: Essential Oil and Supercritical Carbon Dioxide Extract of Grapefruit Peels Formulated for Candida albicans Infections: Evaluation by an in Vitro Model to Study Fungal–Host Interactions
Source: ACS Omega. 2022 Oct 13;7(42):37427–35. doi: 10.1021/acsomega.2c04189 (PMC9608417; doi:10.1021/acsomega.2c04189)
Supplement: Supplementary file 1 — ao2c04189_si_001.pdf [file ao2c04189_si_001.pdf]

Essential oil and supercritical carbon dioxide extract of grapefruit peels formulated for *C. albicans* infections: evaluation by an *in vitro* model to study fungal-host interactions

Burcu Yaldiz[a], Pelin Saglam-Metiner[a], Betul Cakmak[a], Elif Kaya[a], Buse Deliogullari[b], Ozlem Yesil-Celiktas[a,*]

[a]*Department of Bioengineering, Faculty of Engineering, Ege University, 35100, Izmir, Turkey*

[b]*Biomedical Technologies Graduate Programme, Graduate School of Natural and Applied Sciences, Ege University,35100 Bornova, Izmir, Turkey*

*Corresponding author: ozlem.yesil.celiktas@ege.edu.tr

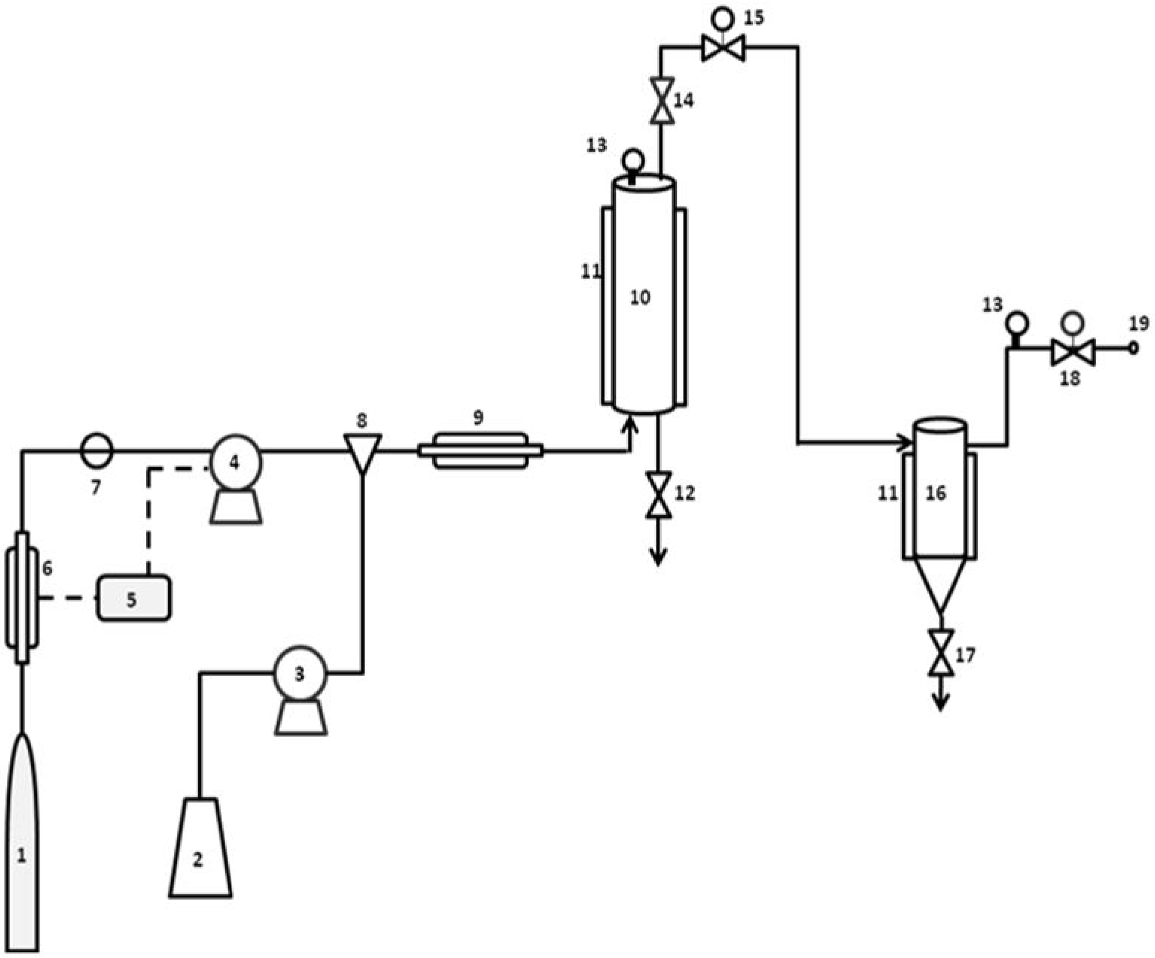

Figure S1. The schematic of SFE 100 System (Thar Instruments, Inc., UK, 2006) (1 - CO_2 supply in, 2 - Co-solvent reservoir, 3 - Co-solvent pump, 4 - CO2 pump, 5 - Cooling bath, 6 - Cooling heat exchanger, 7 - Flow meter, 8 - Mixer, 9 - Heat exchanger, 10 - Extraction vessel, 11 - Heat jacket, 12 - Bleed valve, 13 - Gauge, 14 - On-off valve, 15 – Automated Back Pressure Regulator (BPR), 16 – Fraction collector, 17 – Drain valve, 18 - Manual BPR, 19 – Vent) *Reprinted with permission from Elsevier* [1]

(A)

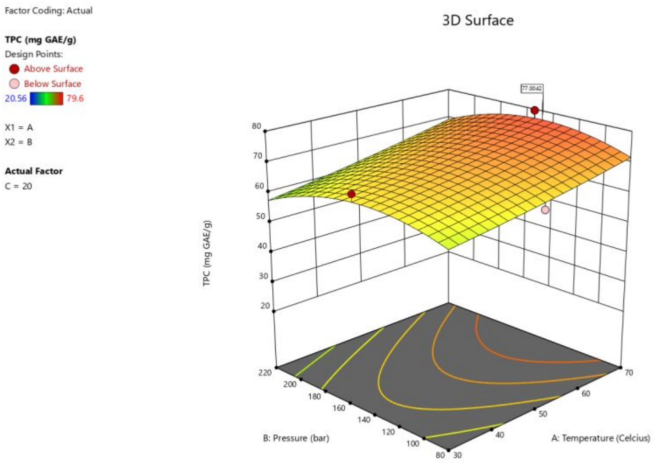

(B)

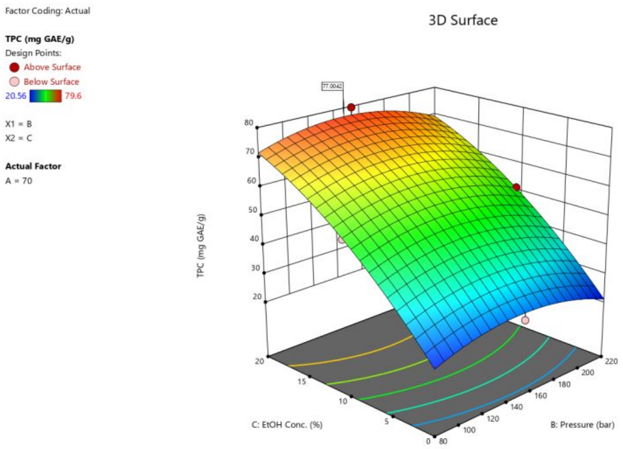

(C)

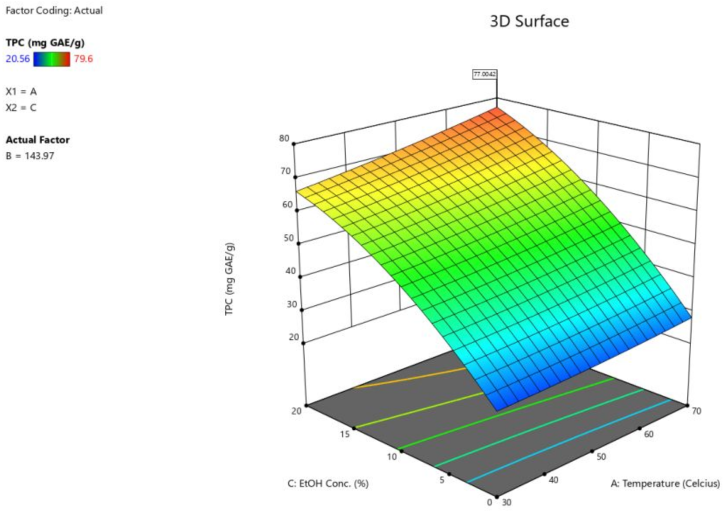

Figure S2. The 3D response surface plots of total phenol contents showing the effects of temperature and pressure at constant optimum co-solvent (20%) (**A**), pressure and co-solvent

at constant temperature (70 °C) (**B**), temperature and co-solvent ratio at constant pressure (150 bar) (**C**).

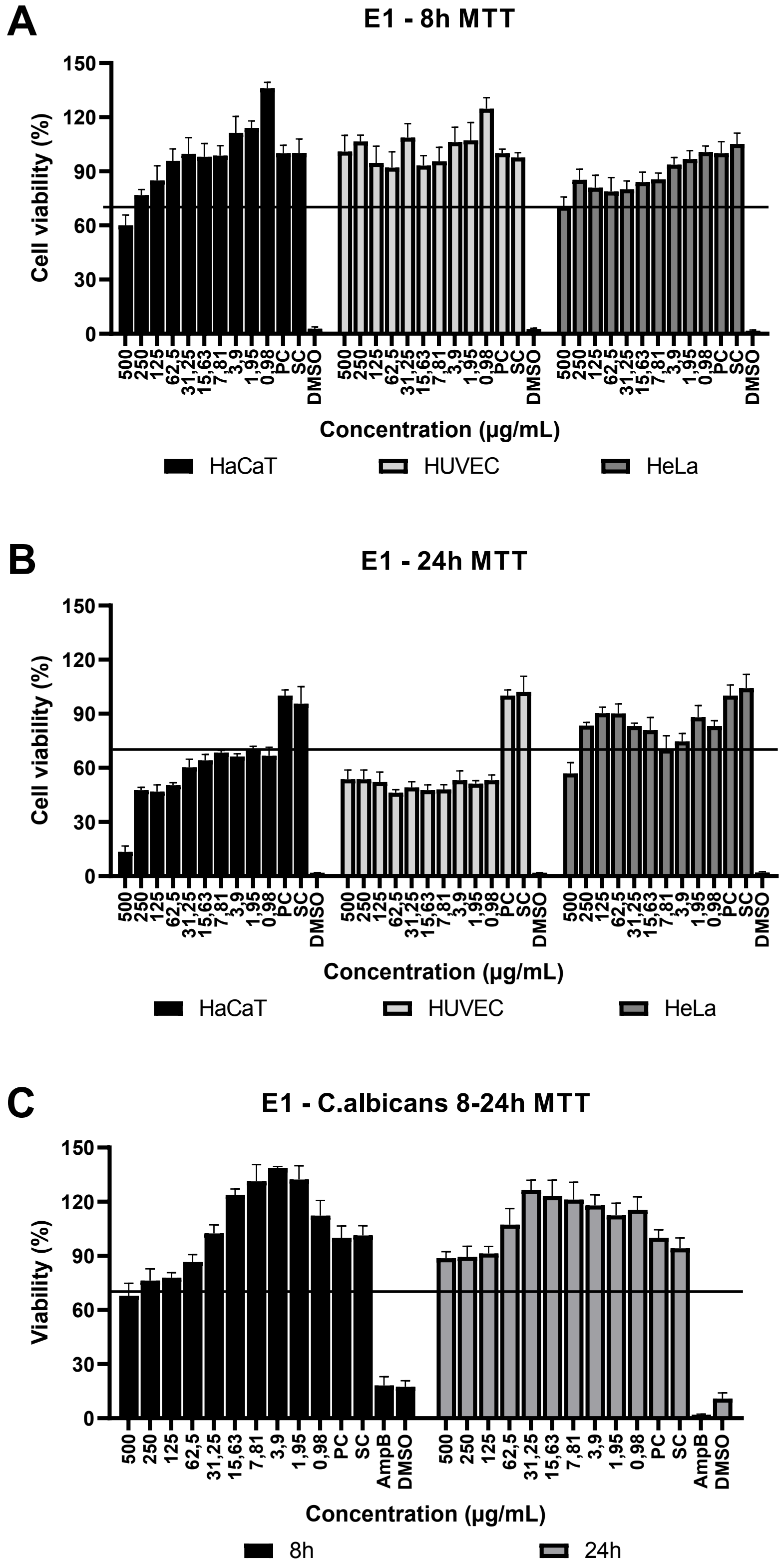

Figure S3. Cell viability results (%) of E1 on on human skin keratinocytes (HaCat), human umbilicord vein endothelial (HUVEC), cervical adenocarcinoma (HeLa) cells at 8 hours (**A**), 24 horus (**B**) and *C.albicans* yeast at 8-24h (**C**).

Reference

(1) Pilavtepe M., Sargin S., Celiktas M.S., Yesil-Celiktas O. (2012) An integrated process for conversion of Zostera marina residues to bioethanol. Journal of Supercritical Fluids, 68, 117-122.
